# Development of a novel UHPLC-MS/MS-based platform to quantify amines, amino acids and methylarginines for applications in human disease phenotyping

**DOI:** 10.1038/s41598-018-31055-8

**Published:** 2018-09-18

**Authors:** Blerina Ahmetaj-Shala, Michael Olanipekun, Abel Tesfai, Niall MacCallum, Nicholas S. Kirkby, Gregory J. Quinlan, Chih-Chin Shih, Ryota Kawai, Sharon Mumby, Mark Paul-Clark, Elizabeth J. Want, Jane A. Mitchell

**Affiliations:** 1 Cardiothoracic Pharmacology, Vascular Biology, National Heart and Lung Institute, Imperial College London, London, SW3 6LY United Kingdom; 2 Department of Surgery and Cancer, Imperial College London, London, SW7 2BB United Kingdom; 3 Critical Care, University College London Hospital, London, NW1 2BU United Kingdom; 4 Respiratory, Airway Disease, National Heart and Lung Institute, Imperial College London, London, SW3 6LY United Kingdom

**Keywords:** Methylarginines, Asymmetric Dimethylarginine (ADMA), Systemic Inflammatory Response Syndrome (SIRS), Ultra-high Performance Liquid Chromatography-tandem Mass, SIRS Score, Biological techniques, Cellular signalling networks, Mass spectrometry

## Abstract

Amine quantification is an important strategy in patient stratification and personalised medicine. This is because amines, including amino acids and methylarginines impact on many homeostatic processes. One important pathway regulated by amine levels is nitric oxide synthase (NOS). NOS is regulated by levels of (i) the substrate, arginine, (ii) amino acids which cycle with arginine and (iii) methylarginine inhibitors of NOS. However, biomarker research in this area is hindered by the lack of a unified analytical platform. Thus, the development of a common metabolomics platform, where a wide range of amino acids and methylarginines can be measured constitutes an important unmet need. Here we report a novel high-throughput ultra-high performance liquid chromatography-tandem mass spectrometry (UHPLC-MS/MS) platform where ≈40 amine analytes, including arginine and methylarginines can be detected and quantified on a molar basis, in a single sample of human plasma. To validate the platform and to generate biomarkers, human plasma from a well-defined cohort of patients before and after coronary artery bypass surgery, who developed systemic inflammatory response syndrome (SIRS), were analysed. Bypass surgery with SIRS significantly altered 26 amine analytes, including arginine and ADMA. Consequently, pathway analysis revealed significant changes in a range of pathways including those associated with NOS.

## Introduction

Amine analysis is increasingly recognised as a valuable approach for the identification of biomarkers and for uncovering new mechanistic pathways associated with disease. One particular amine pathway that is important in all chronic diseases including cancer^1–3^, cardiovascular disease^4–6^ and sepsis^7,8^ is the network that links nitric oxide (NO) with amino acids - including the substrate for NO, L-arginine - and methylarginines, including the substrate-inhibitors asymmetric dimethylarginine (ADMA) and mono-L-methylarginine (L-NMMA). However, measurement of amino acids and related amine containing compounds is relatively difficult, particularly in complex biological matrixes such as plasma, in part due to issues with chromatographic retention and analyte ionisation. Untargeted metabolomics offers the potential to identify changes in amino acids in biological samples as part of a shotgun approach, although current untargeted methods generally provide only relative changes in metabolites. This limitation is particularly relevant when considering amino acid levels where *molar* ratios such as the global arginine bioavailability ratio (GABR), defined as (molar) arginine/[ornithine + citrulline]^9–12^ and the ADMA: arginine ratio are considered more useful than individual levels of arginine or ADMA respectively^9^.

A recent advancement in the metabolomics field was the development of an ultra-high performance liquid chromatography with electrospray ionisation (ESI) coupled to tandem mass spectrometry (UHPLC-MS/MS) platform which we have previously applied to the measurement of amino acids and other amines after derivatisation with 6-aminoquinolyl-N-hydroxysuccinimidyl carbamate (AccQTag Ultra)^13,14^. This assay allows for the simultaneous detection of multiple compounds from parent and fragment ions with high sensitivity and linearity^15^. Using this approach, the accurate detection and quantification of 66 amino acids and biogenic amines was achieved^13,14^. However, whilst this platform provides a means to quantify individual amino acids and amines, crucially the methylarginines were not included in the analysis. This meant that in our previous work quantifying amino acids and methylarginines in samples from patients with sepsis, three separate platforms (UHPLC-MS/MS)^13,14^, LC-MS/MS^14,16^ and ELISA^14,16^ were required. This study sought to address the need for multiple analysis techniques by adopting a similar amino acid UHPLC-MS/MS-based analysis approach but which could detect all of our target amines, including amino acids and methylarginines on one platform in complex matrices, including human plasma. In technical terms, the work described in this report represents a small but very important advancement on our previous method^13,14^.

In order to validate and test the utility of our combined amine platform we have analysed plasma samples from a well-defined cohort of patients before and after cardiopulmonary bypass surgery. We selected this particular clinical scenario because these patients experience a systemic inflammatory response (SIRS) which in many regards recapitulates early inflammatory^17^ events seen in sepsis and that in some cases may even develop into sepsis^18^. Moreover, whilst a full array of amino acids and methylarginines have not been previously measured in samples from cardiopulmonary bypass patients, L-arginine and methylarginine levels have been reported^19^. In this way, measuring plasma samples from the patients provided the opportunity to both test a well-reasoned hypothesis; *‘cardiopulmonary bypass surgery results in a disruption in arginine and related amino acids’* and to generate new hypotheses by using bioinformatics incorporating pathway analysis.

## Results and Discussion

### Method development and validation – identification of specific methylarginine transitions

**Figure 1:**
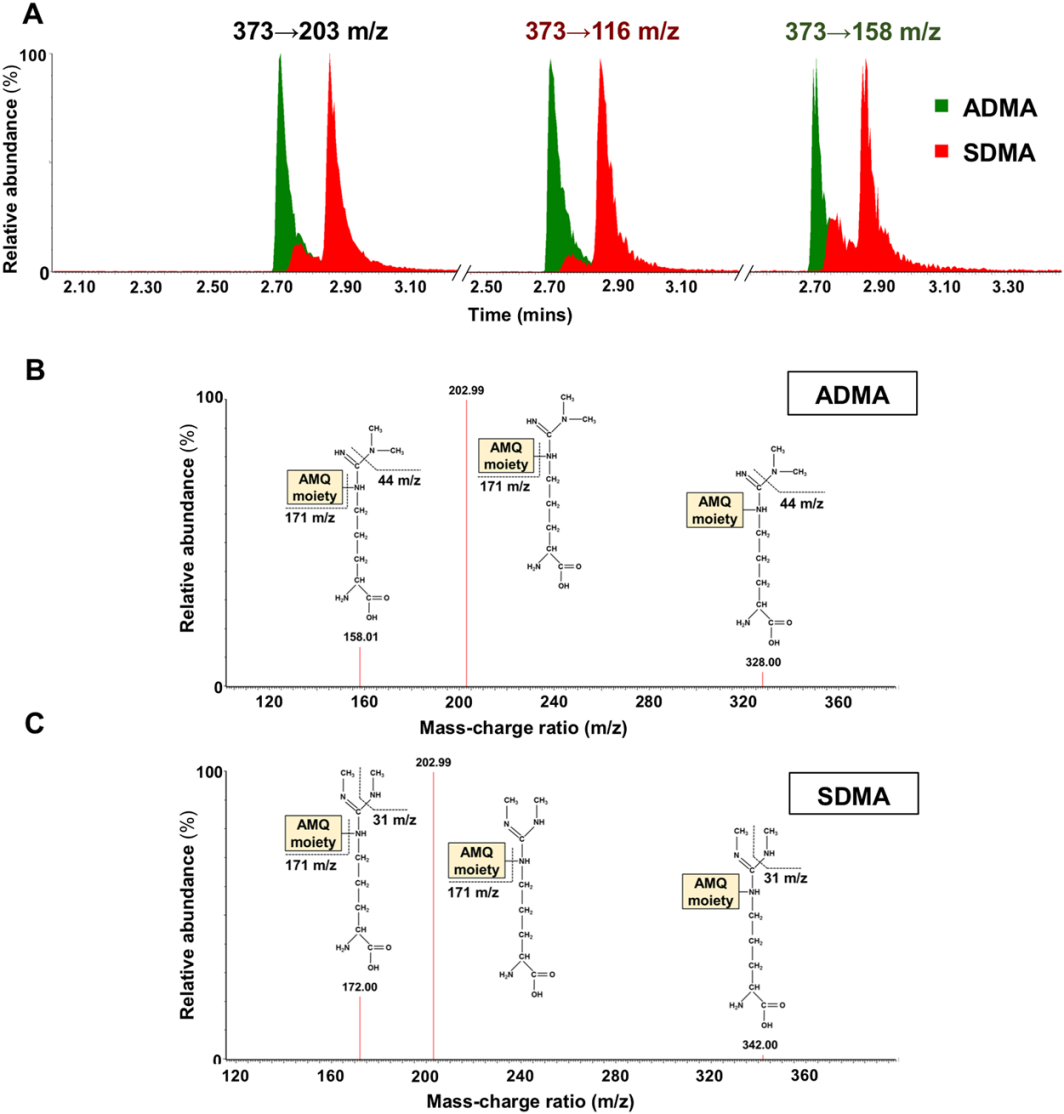
Optimisation of the methylarginine detection method. (**A**) The initial transitions thought to differentiate ADMA (green) and SDMA (red) including the generic transition 373 → 202.99 m/z, and the most abundant transitions seen for ADMA (373 → 158 m/z) and SDMA (373 → 116 m/z), from which a double peak was produced. The specific transitions and fragment ion structures for (**B**) ADMA and (**C**) SDMA are shown with their corresponding m/z. All peaks on the spectra are displayed with their relative abundance (%).

To successfully quantify the new platform of amines, it was first important that the methylarginines, ADMA, L-NMMA and SDMA could be distinguished and identified individually in human plasma samples. Since ADMA and SDMA are structural isomer, they eluted from the UHPLC column with similar retention times of 2.69 and 2.87 minutes, respectively. More importantly, when derivatised with the AccQTag reagent they both had an m/z of 373 and shared a fragment ion with an m/z of 203, representing the molecular weight when underivatised ([M + H] + ion). The change in m/z of a parent ion breaking down to become a fragment ion is described as a ‘transition’ and is often specific to a compound. The first attempt to differentiate these structural isomers used multiple reaction monitoring (MRM) via an automated method (Intellistart; MassLynx) to find the five most abundant transitions for each compound. Of these, some transitions were shared between the compounds and were therefore considered redundant, while others were thought to be specific to either compound (Fig. 1A). Standard solutions were run using the UHPLC-MS/MS method with these transitions and a single peak for ADMA was obtained using the ‘ADMA-specific’ transitions, while SDMA consistently produced a double peak despite using ‘SDMA-specific’ transitions (Fig. 1A). In addition, an ADMA peak could be obtained using ‘SDMA-specific’ transitions, and the same was found for SDMA peaks, which could be obtained with ‘ADMA-specific’ transitions. These observations led us to conclude that these transitions were not specific but shared between the compounds.

**Figure 2:**
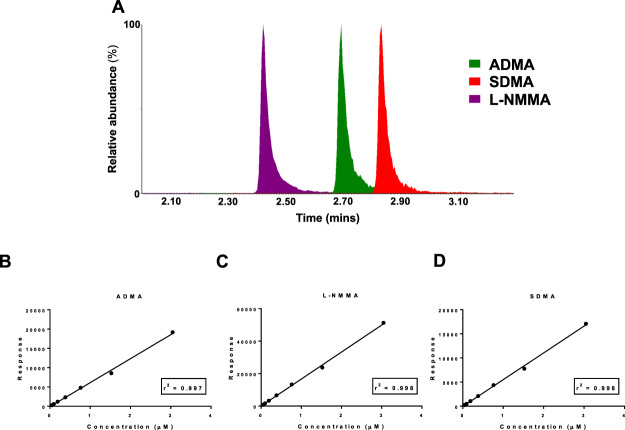
Methylarginine standard curves measured using UHPLC-MS/MS. (**A**) L-NMMA (purple), ADMA (green) and SDMA (red) elute at different retention times (mins) from the UHPLC column. The standard curves for (**B**) ADMA, (**C**) L-NMMA (**D**) and SDMA are displayed with the goodness of fit, represented by the r^2^ values.

In order to solve this problem we adopted a similar approach to one described by Di Gangi and co-workers in 2010^20^ where changes in the methylarginine compound structures could be associated to changes in m/z (transitions). By applying this to our most abundant transitions we could predict the true differentiating transitions based on (i) the structural differences between the structural isomers and (ii) the m/z of the fragment ions that would be specific to each (Fig. 1B,C). We detected ADMA with the 373 → 158 transition, identified previously and differentiated SDMA by its specific 373 → 172 transition (Fig. 1B,C). After deriving these transitions manually, the full range of methylarginine standard solutions (0–3 µM) were run to produce individual chromatograms for ADMA, L-NMMA and SDMA, confirming that the peaks were resolved chromatographically (Fig. 2A). Using the full concentration range, standard curves for each methylarginine were plotted and achieved r^2^ values > 0.997 (Fig. 2B–D). Now, the ADMA, L-NMMA and SDMA parameters could be implemented into the UHPLC-MS/MS assay to create a single platform of 39 analytes (see Supplementary Table 1) for the quantification of methylarginines, along with our previously reported array of amino acids and other amines^13,16^, in a single run.

### Application to human disease

Following the optimisation of the UHPLC-MS/MS platform, we applied the method to the analysis of samples from patients undergoing cardiopulmonary bypass surgery. Samples were collected before and after bypass surgery from a cohort of 20 patients. As we^21^ and others^22^ have shown, cardiopulmonary bypass, by virtue of the invasive nature of the surgery and the requirement for bypass intervention, invariably results in some degree of ‘systemic inflammation’. In this cohort 18 of the patients developed clinically relevant SIRS (see Supplementary Table 2), of which for one patient (patient 1; see Supplementary Table 2) an incomplete sample range was obtained. Thus, samples from 17 patients (patient 2–18), who developed SIRS after bypass, were taken forward for analysis. The array detected a total of 66 amines in human plasma. Our method can quantify 42 individual amines (see Supplementary Table 3). Of these 42, 1-methylhistidine, 3-methylhistidine and glutathione did not produce reliable results due to variabilities in their standard curves (r^2^ value < 0.95), which could be attributed to poor peak shapes when processed.

**Figure 3:**
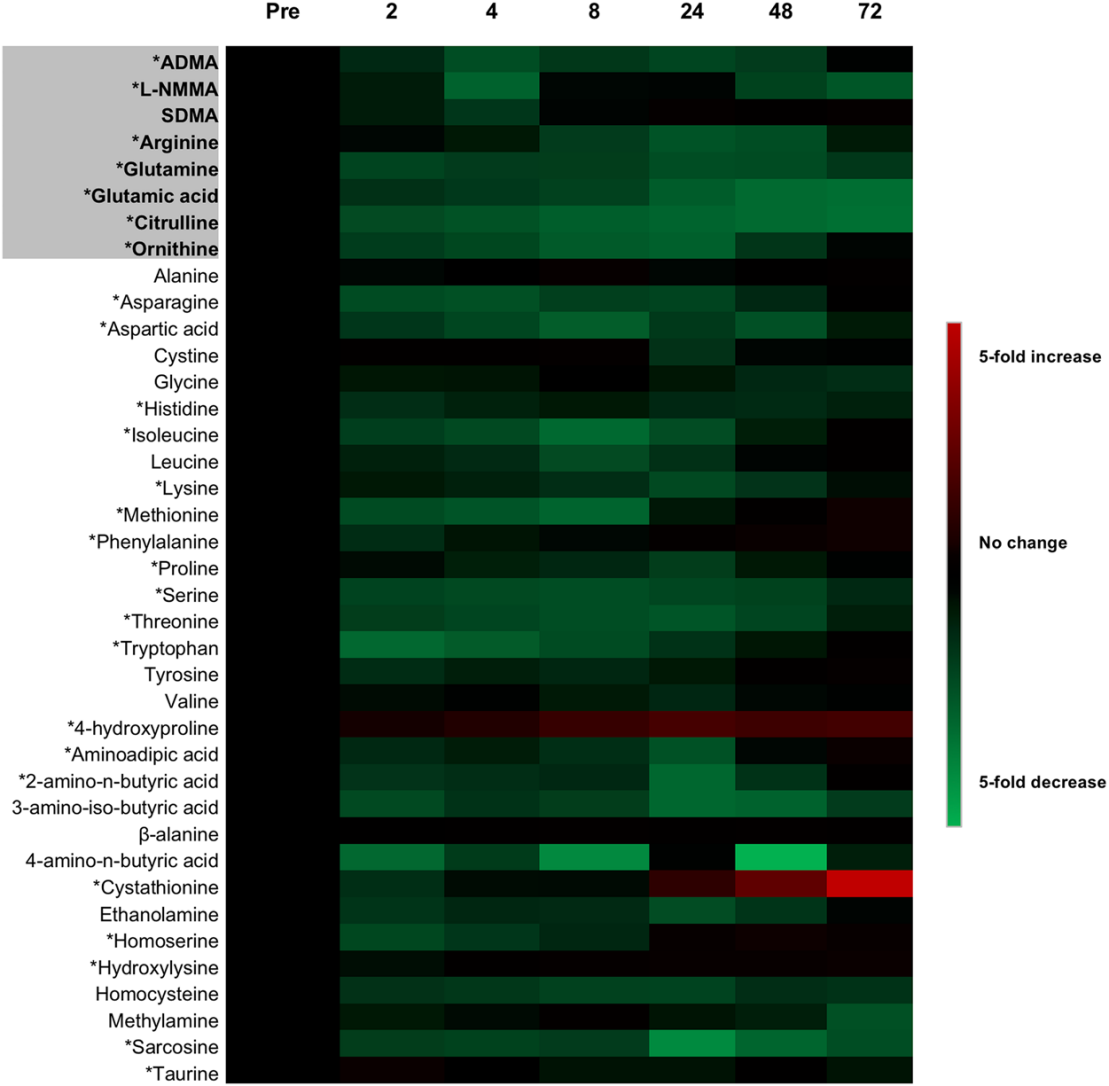
Heat map representation of targeted metabolic profiling of amines and methylarginines in human plasma from patients before and after cardiopulmonary bypass surgery. Amine and methylarginine levels were measured using UHPLC-MS/MS in the plasma of patients before (pre) and at 2–72 h after surgery. Data are ± SEM for n = 17. Data are displayed as a heatmap and were analysed by repeated measures one-way ANOVA with Dunnett’s post-hoc test and Benjamini-Hochberg test with a false discovery rate of 0.05 applied (^*^p < 0.05).

**Figure 4:**
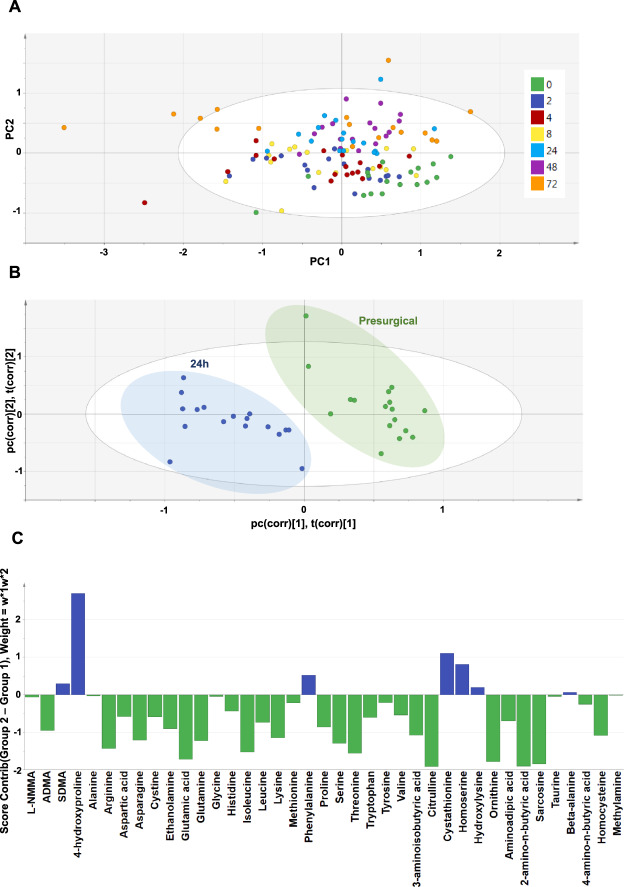
PCA-X scores plot for all patients at every time point and PLS-DA comparing ‘pre-surgical’ and ‘24 h post-surgery’ metabolic profiles. (**A**) The PCA-X scatter plot of all amines in plasma samples from all time points before (0) and after (2–72 h) surgery. Observations are shown as the patient identifiers at each time point and are scattered based on the peak areas for all 39 compounds detected using UHPLC-MS/MS. Data within the ellipse represents the tolerance of Hotelling’s T^2^, revealing outliers as observations present outside of this area. (**B**) The PLS-DA scatter plot shows the deviation of the 24-hour samples (blue) from the pre-surgical sample group (green) via their separate clustering. (**C**) The contributions of each amine to the deviation is represented by the contribution plot, describing the change in amine levels from the pre-surgical (green) to the 24-hour (blue) samples. These changes were assessed based on the weighted differences between the datasets (w*1w*2) in the PLS-DA model. Data shown is for n = 17.

**Figure 5:**
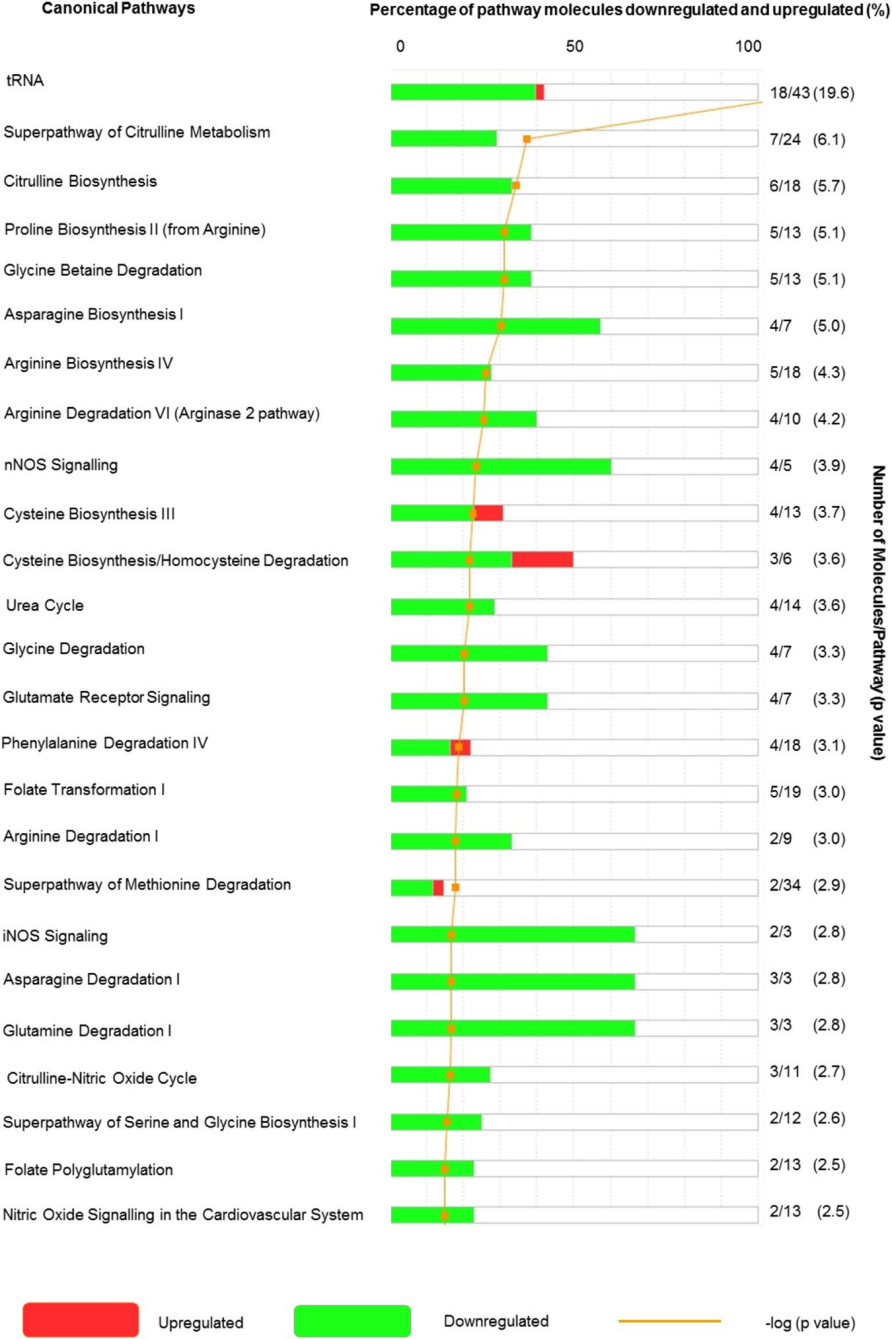
List of the top 25 canonical pathways altered in plasma of patients before and 24 h after surgery. The top 25 canonical pathways altered out of a possible 134. Data are shown as the percentage and number of pathway molecules altered. Ratios were generated for each of the analytes using data for n = 17 patients pre and post-surgery. Data was analysed by Benjamini-Hochberg test with a false discovery rate of 0.05 applied (^*^p < 0.05).

Results from the 39 quantifiable amines (Fig. 3), across all time points (Fig. 3), were processed using principal components analysis (PCA) as an unsupervised approach to establish data clustering and outliers, based on peak areas (Fig. 4A). Ingenuity® Pathway Analysis (IPA) of the total data set revealed significant changes in a range of canonical pathways extracted from IPA software (Fig. 5). The top pathway altered was transfer RNA (tRNA), an adaptor RNA-molecule that acts as a physical link between mRNA and amino acid sequences of proteins. Notably all three NOS pathways were significantly altered by cardiopulmonary bypass surgery (Fig. 5).

Due to the high number of changes seen at 24 h post-surgery, we next performed a partial least squares discriminant analysis (PLS-DA) on all amine data from ‘pre-surgical’ and ‘24 h post-surgery’ samples. PLS-DA confirmed distinct clustering of the two groups, illustrating the presence of a ‘metabolic signature’ associated with bypass surgery based on the global changes in the amine levels (Fig. 4B).

To explore this further, a contribution plot was produced to show how the individual amine levels changed between the pre-surgical and 24-hour post-surgery time points (Fig. 4C). This plot showed SDMA, 4-hydroxyproline and cystathionine levels to be increased, while most of the proteinogenic amino acids were decreased at 24 h, suggesting that these changes drive the deviation between the two groups. These observations were validated using IPA, which linked these alterations to various biological pathways (Fig. 5).

Finally, in order to extract the maximum amount of information from our data, PCA and PLS-DA were also performed using the peak areas of all 42 amines, including 1-methylhistidine, 3-methylhistidine and glutathione (Supplementary Fig. 1). This analysis revealed a relative increase in 1-methylhistidine levels 24 h after surgery.

### Supervised analysis of amino acids/methylarginines of interest and associated ratios

We next performed supervised analysis of the 39 analytes quantified and found that 26 were significantly altered at one or more of the time points post-surgery (Fig. 3; Supplementary Table 3), with most of them showing a reduction in levels (Fig. 3; Supplementary Table 3). The exceptions were 4-hydroxyproline, cystathionine and phenylalanine which increased after surgery (Fig. 3; Supplementary Table 3). 4-hydroxyproline is an important amine constituent of several extracellular matrix proteins and is used as a marker of collagen degradation^23^. As such, the increased levels of 4-hydroxyproline seen in patient samples is consistent with the nature of inflammatory events occurring in this type of major surgery, whilst cystathionine is a substrate for the gaseous mediator hydrogen sulphide and also a precursor for glutathione production. Parallels are often drawn between hydrogen sulphide and NO, both of which have dual protective and inflammatory roles depending upon the concentrations generated and the local environment^24,25^. Cystathionine and glutathione (not shown) are linked in the *‘methionine and glutathione metabolic pathway’* with cysteine as an intermediate. However, cystine (a stable oxidation product of cysteine) levels did not increase in our samples suggesting that the increase in cystathionine is not a consequence of general up-regulation in methionine metabolism. Whilst the increases in phenylalanine were relatively modest, our results are consistent with findings showing increased plasma levels of phenylalanine in patients with trauma and sepsis^26^. Our findings that increased phenylalanine was not accompanied by increases in tyrosine suggests a reduction in phenylalanine hydrolase activity consistent with ‘phenylalanine degradation’ and ‘tyrosine biosynthesis’ pathways being highlighted in the IPA analysis of samples 24 h after surgery (Fig. 5). Of the pathways reduced following bypass surgery all 8 (ADMA, L-NMMA, SDMA, arginine, glutamine, glutamic acid, citrulline and ornithine) of the amino acids and methylarginines associated (directly or indirectly) with the NOS pathway were included (Fig. 5). To explore the impact and of these changes in regard to NOS activity supervised analysis of these analytes was performed.

### Arginine and related amino acids and associated ratios: arginine: ornithine and GABR (arginine/(ornithine + citrulline))

L-arginine availability is not always well-represented solely by the concentration of arginine in plasma. This is because arginine cycles with citrulline and ornithine, and because glutamate and glutamine are metabolised to ornithine. Glutamine, which is a predictor of mortality in critically ill patients^27^, is additionally important to consider in terms of arginine and the NOS pathway. Not only can arginine be generated from glutamine but as we have shown^28–30^, glutamine also acts as a brake on endogenous arginine generation via the urea cycle.

**Figure 6:**
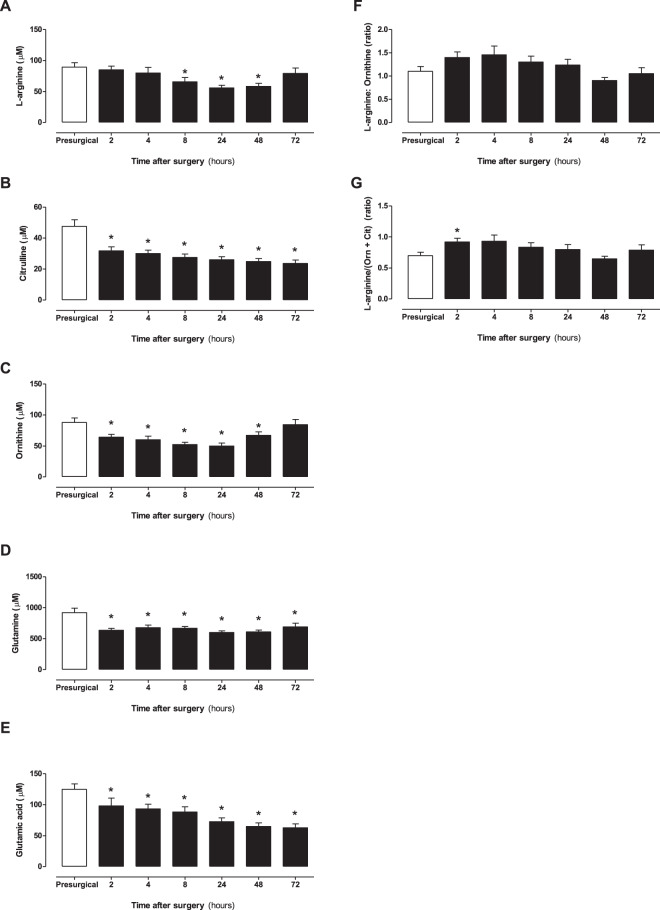
The concentration of NO related amines and associated ratios in human plasma from patients before and after surgery. (**A**) L-arginine (**B**) citrulline, (**C**) ornithine, (**D**) glutamine, (**E**) glutamic acid, (**F**) arginine: ornithine ratio and (**G**) global arginine global arginine bioavailability ratio (GABR; L-arginine/(ornithine + citrulline)) are also represented. Data are mean ± SEM for n = 17 patients and were analysed using a repeated measures one-way ANOVA followed by a Dunnett’s post-test comparing all post-surgery time points to the pre-surgical control (^*^p < 0.05).

In each case, arginine, citrulline, ornithine, glutamine and glutamic acid all decreased for at least two consecutive time points after surgery, although the duration of decrease differed between these amino acids (Fig. 6). These changes impacted on the arginine: ornithine ratio; a biomarker of arginase activity and a predictor of a wide range of clinical outcomes in a number of diseases^31^ including sickle cell^32^ and on GABR (arginine/(ornithine + citrulline)) (Fig. 6). GABR is a biomarker thought to more accurately reflect L-arginine bioavailability than arginine concentration alone, since it considers the impact of renal function and *de novo* arginine biosynthesis from citrulline. Thus, in bypass patient samples both the arginine: ornithine ratio and GABR were transiently increased 2–4 h after bypass, returning to control levels by 48 h after surgery (Fig. 6). Whilst the actual meaning of GABR to functional arginine availability within cells can only be inferred, this observation shows that whilst arginine levels are stable in the first few hours after surgery, its relative availability (GABR) may increase at these time points potentially via reduced arginase activity directly following surgery. The reduction in arginine seen at 8, 24 and 48 h after surgery was not accompanied by changes in arginine: ornithine ratio, ruling out increased arginase as an explanation leaving increased NOS activity within tissues as a potential reason for arginine depletion at these time points.

### Methylarginines and methylarginine: arginine ratios

**Figure 7:**
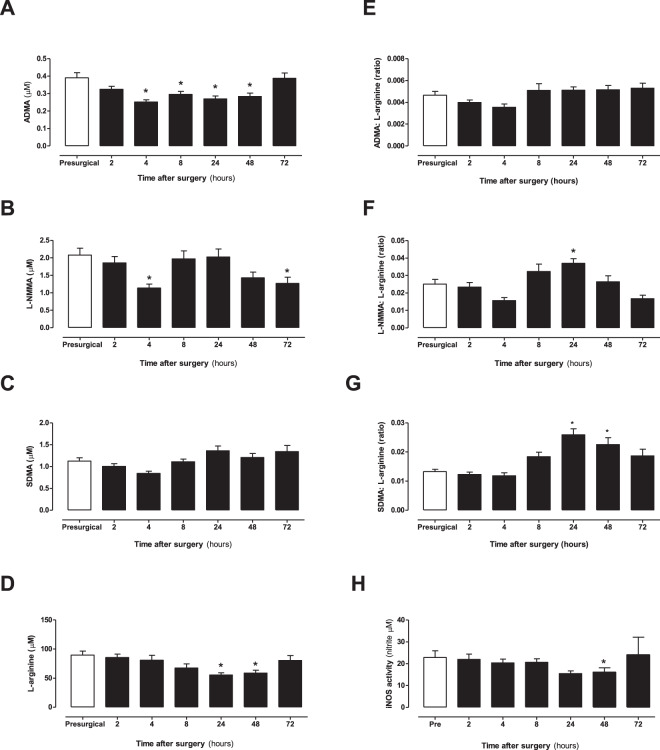
The concentration of L-arginine, ADMA, L-NMMA and SDMA and associated ratios and iNOS supporting activity in human plasma from patients before and after surgery. (**A**) ADMA, (**B**) L-NMMA, (**C**) SDMA, (**D**) L-arginine, (**E**) ADMA: L-arginine ratio, (**F**) L-NMMA: L-arginine ratio, (**G**) SDMA: L-arginine ratio in plasma and (**H**) iNOS supporting activity of plasma applied to LPS activated J774 macrophages. Data are mean ± SEM for n = 17 patients before and after surgery and were analysed using a repeated measures one-way ANOVA followed by a Dunnett’s post-test comparing all post-surgery time points to the pre-surgical control (^*^p < 0.05).

**Figure 8:**
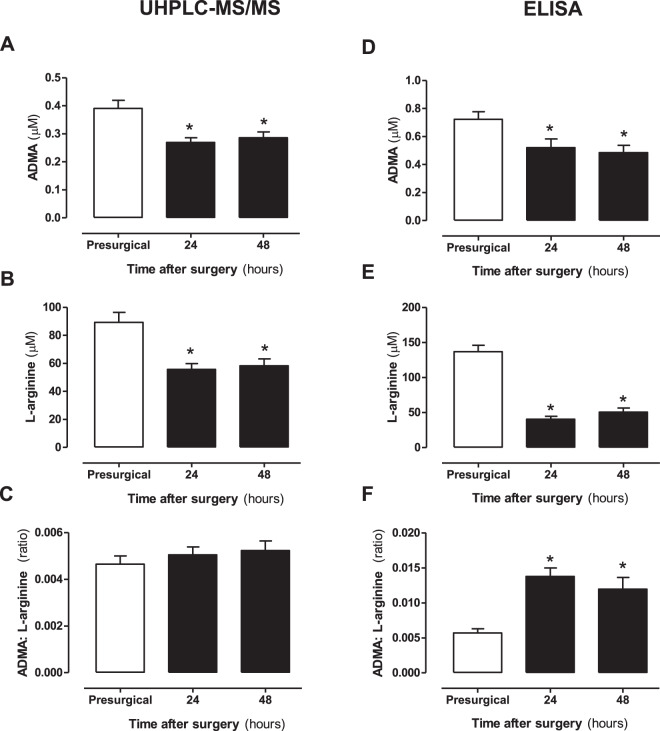
Validation of ADMA and L-arginine concentration using UHPLC-MS and ELISA. (**A**–**C**) UHPLC-MS/MS and (**D**–**F**) ADMA/L-arginine ELISA. Data are mean ± SEM for n = 17 patients. Data were analysed using a paired one-way ANOVA followed by a Dunnett’s post-test (^*^p < 0.05).

Methylarginines are produced after arginine residues in proteins are methylated by PRMT1 and following subsequent protein breakdown^33^. Methylarginines are removed from the circulation by metabolism by DDAH and AGXT2 enzymes and by excretion in the kidney^33^. ADMA is the most studied methylarginine and elevated plasma levels have been associated with cardiovascular disease^33^, sepsis^14,33^ and inhibition of cyclooxygenase-2 by pain medications^16^. Despite being at least as potent as ADMA (Supplementary Fig. 2), L-NMMA has frequently been regarded as physiologically irrelevant due to its relatively low plasma levels^34^. However, our analytical method shows that plasma L-NMMA concentrations are actually substantially higher than ADMA at all assessed time points (Fig. 7). SDMA is generally considered to be biologically inert, although some studies have suggested that it may compete with arginine for transport into cells. We found that similarly to arginine, ADMA and L-NMMA, but not SDMA, were reduced at two or more time points after surgery. The concerted changes in arginine and methylarginine concentrations did not significantly alter ADMA: arginine ratio but did increase L-NMMA: arginine and SDMA: arginine ratios after bypass surgery. In order to test the impact of arginine and methylarginine levels in plasma after cardiopulmonary bypass surgery we analysed the ‘iNOS supporting activity’ of the plasma on LPS-activated mouse macrophages using a bioassay system that we have recently developed and validated^14^. iNOS activity was reduced at 24 and 48 h after surgery which coincides with reduced arginine and increased L-NMMA: arginine ratio (Fig. 7). These findings validate biologically the changes in arginine and methylarginines in plasma after bypass measured on our platform. Whilst MS/MS is commonly used to measure methylarginines and arginine concentrations, there are commercially available ELISAs for the measurement of ADMA and arginine. In order to cross-validate our platform and one commonly used ELISA we performed a subset analysis using both techniques (Fig. 8).

### Arginine and methylarginine validation using ELISA

L-arginine and ADMA levels measured in plasma by both UHPLC-MS/MS or ELISA showed similar relative concentrations and patterns of change after bypass surgery (Fig. 8). However, it should be noted that subtle differences in the relative arginine concentrations as determined by ELISA resulted in an increased ADMA: arginine ratio being recorded in plasma samples after surgery using this assay. The ELISA kit, which is a combined platform for ADMA and arginine, declares cross reactivity (%) of ADMA with arginine (0.03), SDMA (0.05) and L-NMMA (1.93) and of arginine with ADMA (<0.37), homoarginine (2.92) and SDMA (0.8). These (and potentially other) cross-reactivities could explain the higher versus lower levels of arginine recorded by the ELISA pre and post bypass respectively. These findings show that whilst ELISAs can provide very useful indicators of arginine and ADMA levels, their accuracy for determining absolute concentrations may be confounded by other constituents in plasma.

## Conclusion

Metabolomics is a relatively new and continuously developing field of study able to provide biological insight into many areas of health and disease. In the case of amino acids and methylarginines, it is important that absolute concentrations are determined in order to construct meaningful conclusions related to biomarkers, mechanisms and pathway associations. In this study we have devised a single platform whereby a full range of amino acids, amines and methylarginines can be measured in one sample on a single platform. Our new platform proved useful in phenotyping patients after cardiopulmonary bypass surgery with SIRS, providing novel insights into metabolic changes. Based on the extensive involvement of amines involved in the NO pathways in all areas of human health and disease, this platform is likely to have extensive utility within the basic science and clinical community.

## Methods

### Chemicals and reagents

All analyte standards including methylarginines (listed in Supplementary Table 1) were obtained from Sigma-Aldrich (Gillingham, U.K.). Isotopically labelled amino acids for use as internal standards (IS) were from Cambridge Isotope Laboratories (MA, U.S.A.) or QMX Laboratories (Essex, U.K.). Water, Optima^TM^ LC/MS grade was obtained from Fisher Scientific (Leicester, U.K.), LC-MS grade solvents and formic acid were from Sigma-Aldrich (Gillingham, U.K.) and the AccQTag Ultra reagents including borate buffer from Waters Corporation (Milford, MA, U.S.A.). All UHPLC-MS/MS instrumentation was also obtained from Waters Corporation (Milford, MA, U.S.A.). The ADMA-L-arginine ELISA kit was obtained from DLD Diagnostika GmbH (Hamburg, Germany).

### Clinical study

Plasma samples were collected into anticoagulant heparin vacutainers from patients (n = 18; 14 male and 4 female; 66 ± 11.6 years) before cardiopulmonary bypass surgery (pre-surgical) and at 2, 4, 8, 24, 48 and 72 h post-surgery at Royal Brompton Hospital, UK. The SIRS status of each patient was determined following surgery as the SIRS score (SIRS score = number of SIRS criteria × number of hours (h) presenting within 24 h in the ICU). In order to be classified as having SIRS, patients needed to have a SIRS score ≥1 (Supplementary Table 1). This study was approved by a Research Ethics Committee at Royal Brompton Hospital (RBH 01-15 152), Imperial College London (RBH 00-062). All volunteers gave written informed consent before entering the study. All methods were performed in accordance with the relevant guidelines and regulations.

### Analytical procedure

#### Preparation of quality control, amino acid and methylarginine calibration solutions

A standard stock solution of amino acids and quality control solutions were prepared as described previously^13,14^. In addition, methylarginine (ADMA, L-NMMA and SDMA) stock solutions (10 mM) were prepared and diluted with 50:50 water/methanol (v/v) to give concentrations of 0, 0.05, 0.1, 0.19, 0.38, 0.76, 1.5 and 3 μM. All standards were run at the beginning and end of the sample batch.

### Sample preparation

To remove any protein, plasma (10 µl) was mixed with LCMS grade water (10 µl) and internal standard solution (10 µl; 2 µg/ml) was added to 1% formic acid in isopropanol (40 µl) and incubated at −20 °C for 20 mins. The samples were centrifuged at 10,000 × g for 10 mins and the supernatant (10 µl) transferred to a new tube for derivatisation (see below).

### Derivatisation

To each sample, borate buffer (70 µl; pH 8.6) was added, mixed briefly and centrifuged at 2,000 × g for 2 mins before the addition of the AccQTag Ultra derivatising reagent (20 µl) for derivatisation at 55 °C for 10 mins. Samples were then transferred to 96-well plates (Waters Corporation, UK) and diluted 1:20 (plasma) or 1:100 (standards) in LCMS grade water.

### Detection of analytes using UHPLC-MS/MS

Analytes were detected as described previously^14^. Briefly, UHPLC-MS/MS analysis with an integrated Xevo TQ-S tandem quadrupole mass spectrometer (Waters Corporation, UK) and a reversed-phase UHPLC separation was performed using a HSS T3 (2.1 × 150 mm, 1.8 µm) column (Waters Corporation, UK) and mobile phases of 0.1% formic acid in water (Fisher Chemical, USA) and 0.1% formic acid in acetonitrile (Riedel-de Haën, USA). In addition to the amine transitions already established, specific transitions were set for the identification and quantification of ADMA (373 → 158), SDMA (373 → 172) and L-NMMA (359 → 188.9).

### Data analyses and bioinformatics

UHPLC-MS/MS data were collected and visualised using MassLynx V4.1 (Waters Corporation) and the analyte peaks analysed using TargetLynx V4.1 (Waters Corporation). The ratio of healthy donor and patient amine measurements were measured (IPA, Qiagen Redwood City, www.qiagen.com/ingenuity) and the association between analytes and canonical pathways tested by the Benjamini-Hochberg test with a false discovery rate of 0.05. Soft-independent modelling of class analogy V14.1 (SIMCA) was used to model principal components analysis (PCA) in an unsupervised (PCA-X) and supervised way, with relationships between patients at different time points set across PC1-3. All data were log transformed and mean-centred for PCA. Supervised analysis is described as partial least squares-discriminatory analysis (PLS-DA). PLS-DA was used to compare 24 h samples and pre-surgical samples. Ellipses were formed from Hotelling’s T^2^ test with a 95% confidence limit (see Supplementary Fig. 3 and Supplementary Table 4).

### ADMA and L-arginine ELISA

ADMA and L-arginine levels in clinical samples were determined using a commercially available ELISA (DLD Diagnostika, Germany), as described previously^14^.

### Heat map construction

UHPLC-MS/MS amine measurements from blood donations at different time points were normalised to their co-responding pre-surgical values. Fold changes are shown by pictographic scale representing a five-fold increase (red) or decrease (green).

### iNOS supporting bioassay

iNOS supporting activity was measured using a novel mouse macrophage bioassay recently reported by us^14^. Briefly, RAW 264.7 mouse macrophages (ATCC, USA), were cultured using Dulbecco’s Modified Eagle’s Medium (Sigma-Aldrich, UK) supplemented with 2mM L-glutamine (Sigma Aldrich, UK), nonessential amino acids (Invitrogen, UK) and penicillin-streptomycin (Sigma Aldrich, UK) at 5% CO_2_ and 37 °C. At confluence, cells were scraped and spun at 400 relative centrifugal force for 5 minutes. 10% filtered foetal bovine serum (LabTech, UK) was included only when culturing. For the iNOS supporting bioassay, media was replaced with neat (100%) human plasma from patients before and at different times after cardiopulmonary bypass – this was added directly to RAW 264.7 cells. Cells were then stimulated to express iNOS with LPS (1 μg/ml) for 24 h. Plasma was then removed and immediately assays nitrite levels as a marker of iNOS activity. Nitrite was measured using the Griess assay as we have described previously^14^.

### Statistical analysis

UHPLC-MS/MS and ELISA data were expressed as the mean ± S.E.M for n donors. Unless otherwise stated, statistical tests were performed using GraphPad Prism v6 (GraphPad Inc., UK) and defined in figure legends. Statistical significance was noted when *p < 0.05.

### Electronic supplementary material

Supplementary information

## Data Availability

All data generated or analysed during this study are included in this published article (and its supplementary information files).
