# Author Correction: Development of a novel UHPLC-MS/MS-based platform to quantify amines, amino acids and methylarginines for applications in human disease phenotyping

**DOI:** 10.1038/s41598-024-59923-6

**Published:** 2024-05-23

**Authors:** Blerina Ahmetaj-Shala, Michael Olanipekun, Abel Tesfai, Niall MacCallum, Nicholas S. Kirkby, Gregory J. Quinlan, Chih-Chin Shih, Ryota Kawai, Sharon Mumby, Mark Paul-Clark, Elizabeth J. Want, Jane A. Mitchell

**Affiliations:** 1 Cardiothoracic Pharmacology, Vascular Biology, National Heart and Lung Institute, Imperial College London, London, SW3 6LY UK; 2 Department of Surgery and Cancer, Imperial College London, London, SW7 2BB UK; 3 Critical Care, University College London Hospital, London, NW1 2BU UK; 4 Respiratory, Airway Disease, National Heart and Lung Institute, Imperial College London, London, SW3 6LY UK

Correction to: *Scientific Reports* 10.1038/s41598-018-31055-8, published online 18 September 2018

This Article contains errors in the Results and Discussion section, Figures 3, 4 and 7, the legends of Figures 2 and 7, as well as the Supplementary Information.

In the Results and Discussion section, under the subheading ‘Application to human disease’,

“The array detected a total of 66 amines in human plasma. Our method can quantify 42 individual amines (see Supplementary Table 3). Of these 42, 1-methylhistidine, 3-methylhistidine and glutathione did not produce reliable results due to variabilities in their standard curves (r2 value < 0.95), which could be attributed to poor peak shapes when processed.”

should read:

“The array detected a total of 65 amines in human plasma. Our method can quantify 41 individual amines (see Supplementary Table 3). Of these 41, 1-methylhistidine, 3-methylhistidine and glutathione did not produce reliable results due to variabilities in their standard curves (r^2^ value < 0.95), which could be attributed to poor peak shapes when processed.”

“Results from the 39 quantifiable amines (Fig. 3), across all time points (Fig. 3), were processed using principal components analysis (PCA) as an unsupervised approach to establish data clustering and outliers, based on peak areas (Fig. 4A).”

should read:

“Results from the 38 quantifiable amines (Fig. 3), across all time points (Fig. 3), were processed using principal components analysis (PCA) as an unsupervised approach to establish data clustering and outliers, based on peak areas (Fig. 4A).”

“Finally, in order to extract the maximum amount of information from our data, PCA and PLS-DA were also performed using the peak areas of all 42 amines, including 1-methylhistidine, 3-methylhistidine and glutathione (Supplementary Fig. 1).”

should read:

“Finally, in order to extract the maximum amount of information from our data, PCA and PLS-DA were also performed using the peak areas of all 41 amines, including 1-methylhistidine, 3-methylhistidine and glutathione (Supplementary Fig. 1).”

Under the subheading ‘Supervised analysis of amino acids/methylarginines of interest and associated ratios’,

“We next performed supervised analysis of the 39 analytes quantified and found that 26 were significantly altered at one or more of the time points post-surgery (Fig. 3; Supplementary Table 3), with most of them showing a reduction in levels (Fig. 3; Supplementary Table 3).”

should read:

“We next performed supervised analysis of the 38 analytes quantified and found that 25 were significantly altered at one or more of the time points post-surgery (Fig. 3; Supplementary Table 3), with most of them showing a reduction in levels (Fig. 3; Supplementary Table 3).”

“Of the pathways reduced following bypass surgery all 8 (ADMA, L-NMMA, SDMA, arginine, glutamine, glutamic acid, citrulline and ornithine) of the amino acids and methylarginines associated (directly or indirectly) with the NOS pathway were included (Fig. 5).”

should read:

“Of the pathways reduced following bypass surgery amino acids and methylarginines associated (directly or indirectly) with the NOS pathway were included (Fig. 5).”

Under the subheading ‘Methylarginines and methylarginine: arginine ratios’, the following sentence should be deleted: “However, our analytical method shows that plasma L-NMMA concentrations are actually substantially higher than ADMA at all assessed time points (Fig. 7).”

“We found that similarly to arginine, ADMA and L-NMMA, but not SDMA, were reduced at two or more time points after surgery. The concerted changes in arginine and methylarginine concentrations did not significantly alter ADMA: arginine ratio but did increase L-NMMA: arginine and SDMA: arginine ratios after bypass surgery.”

should read:

“We found that similarly to arginine, ADMA but not SDMA, was reduced at two or more time points after surgery. The concerted changes in arginine and methylarginine concentrations did not significantly alter ADMA: arginine ratio but did increase SDMA: arginine ratios after bypass surgery.”

“iNOS activity was reduced at 24 and 48 h after surgery which coincides with reduced arginine and increased L-NMMA: arginine ratio (Fig. 7).”

should read:

“iNOS activity was reduced at 24 and 48 h after surgery which coincides with reduced arginine (Fig. 7).”

In the legend of Figure 2, the following sentence should be added: “It is important to note that LNMMA and homoarginine share the same molecular weight and MS parameters. Under the experimental conditions reported in this paper, they can only be distinguished using chromatographic retention time.”

In addition, in Figures 3, 4 and 7, the analysis of LNMMA should be removed. The correct Figures and accompanying legends appear below.

**Figure 3:**
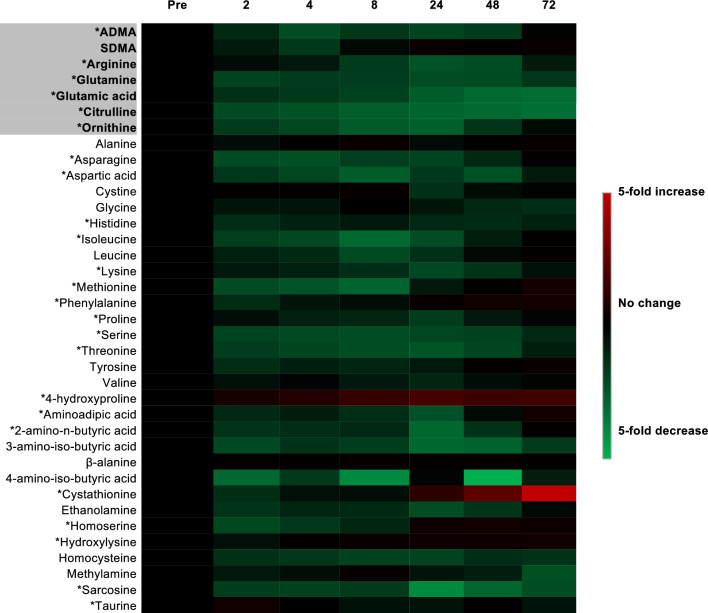
Heat map representation of targeted metabolic profiling of amines and methylarginines in human plasma from patients before and after cardiopulmonary bypass surgery. Amine and methylarginine levels were measured using UHPLC-MS/MS in the plasma of patients before (pre) and at 2–72 h after surgery. Data are ± SEM for n = 17. Data are displayed as a heatmap and were analysed by repeated measures one-way ANOVA with Dunnett’s post-hoc test and Benjamini–Hochberg test with a false discovery rate of 0.05 applied (*p < 0.05).

**Figure 4:**
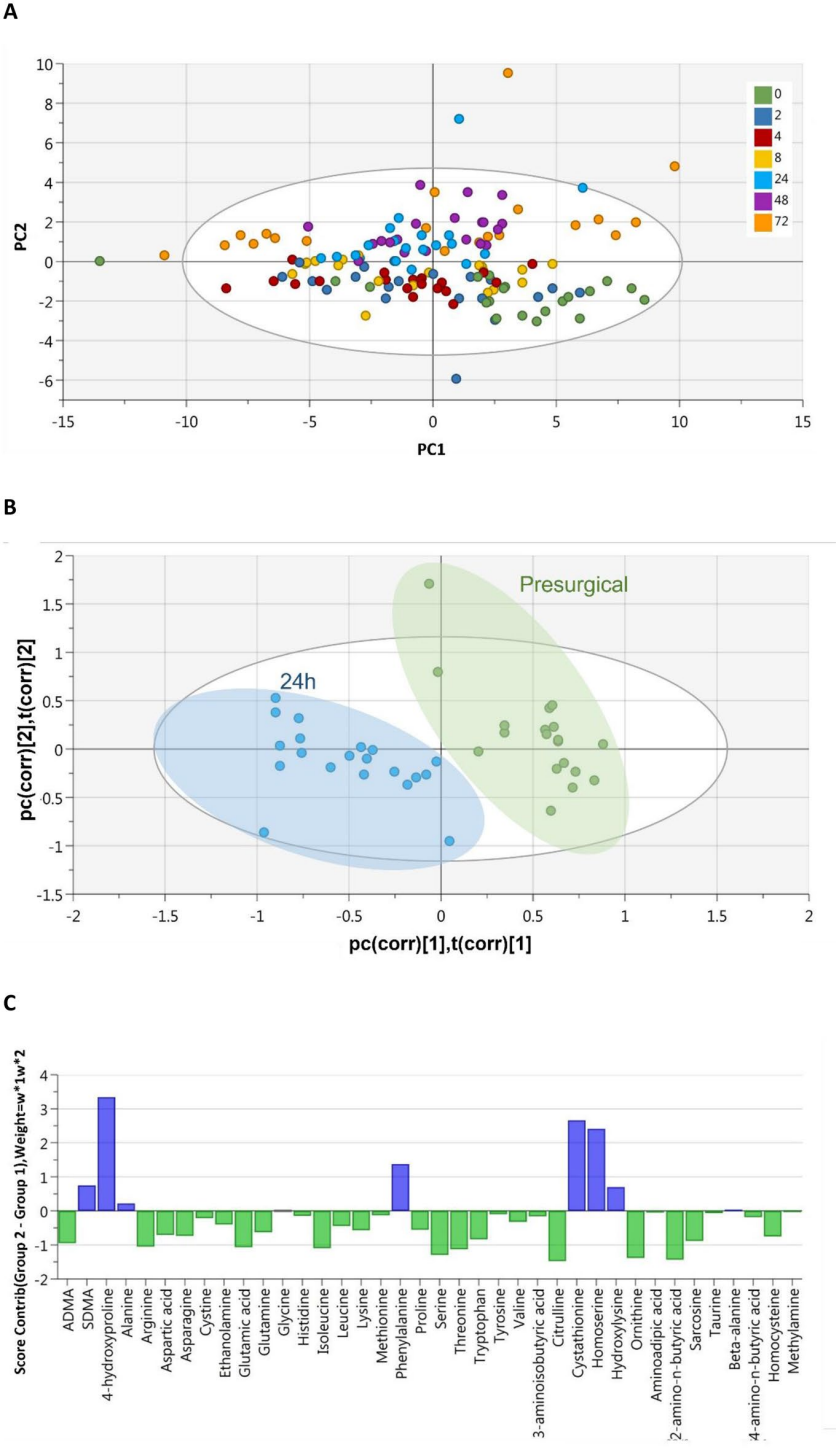
PCA-X scores plot for all patients at every time point and PLS-DA comparing ‘pre-surgical’ and ‘24  h post-surgery’ metabolic profiles. (**A**) The PCA-X scatter plot of all amines in plasma samples from all time points before (0) and after (2–72  h) surgery. Observations are shown as the patient identifiers at each time point and are scattered based on the peak areas for compounds detected using UHPLC-MS/MS. Data within the ellipse represents the tolerance of Hotelling’s T^2^, revealing outliers as observations present outside of this area. (**B**) The PLS-DA scatter plot shows the deviation of the 24-h samples (blue) from the pre-surgical sample group (green) via their separate clustering. (**C**) The contributions of each amine to the deviation is represented by the contribution plot, describing the change in amine levels from the pre-surgical (green) to the 24-h (blue) samples. These changes were assessed based on the weighted differences between the datasets (w*1w*2) in the PLS-DA model. Data shown is for n = 17.

**Figure 7:**
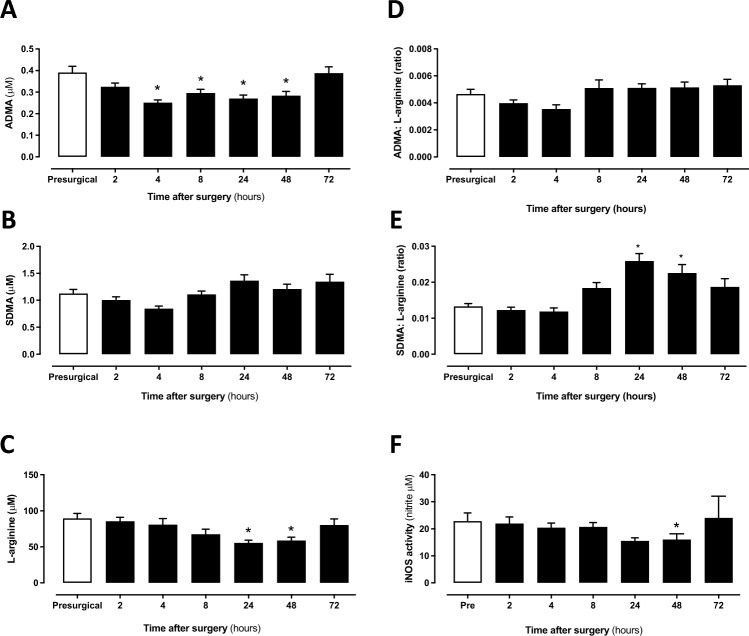
The concentration of L-arginine, ADMA, and SDMA and associated ratios and iNOS supporting activity in human plasma from patients before and after surgery. (**A**) ADMA, (**B**) SDMA, (**C**) L-arginine, (**D**) ADMA: L-arginine ratio, (**E**) SDMA: L-arginine ratio in plasma and (**F**) iNOS supporting activity of plasma applied to LPS activated J774 macrophages. Data are mean ± SEM for n = 17 patients before and after surgery and were analysed using a repeated measures one-way ANOVA followed by a Dunnett’s post-test comparing all post-surgery time points to the pre-surgical control (*p < 0.05).

In the Supplementary Information, the analysis of LNMMA should be removed from Supplementary Figure 1 as well as Supplementary Tables 3 and 4. The correct Supplementary Figure 1 and Supplementary Tables 3 and 4 as well as accompanying legends appear below.

**Figure:**
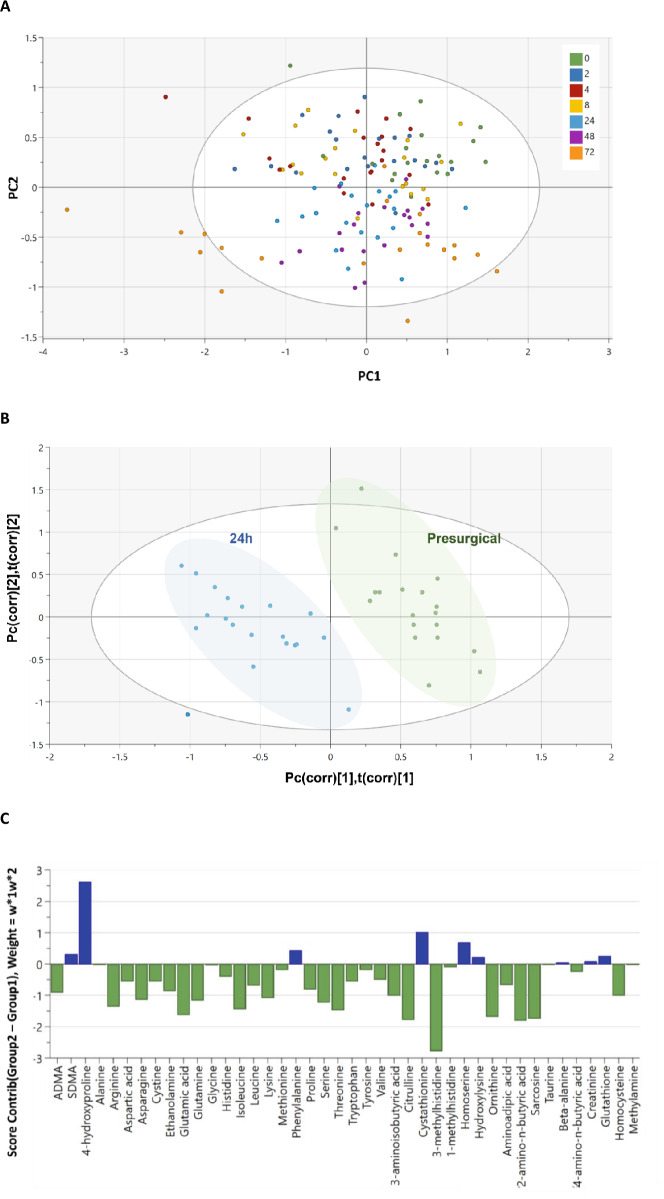
Figure S1. PCA-X and PLS-DA score plots for all patients at every time point, including previously excluded amines 1-methylhistidine, 3-methylhistidine and glutathione. (**A**) The PCA-X scatter plot of all amine peak areas obtained from plasma samples collected before (0) and after (2–72 hours) surgery. Observations are shown as the patient identifiers at each time point and are scattered based on the data for 41 compounds detected using UHPLC-MS/MS. Data within the ellipse represents the tolerance of Hotelling’s T2, revealing outliers as observations present outside of this area. (**B**) The PLS-DA scatter plot shows the deviation of the 24-hour samples (blue) from the pre-surgical sample group (green) via their separate clustering. (**C**) The contributions of each amine to the deviation is represented by the contribution plot, describing the change in amine levels from the pre-surgical (green) to the 24-hour (blue) samples. These changes were assessed based on the weighted differences between the datasets (w*1w*2) in the PLS-DA model. Data shown is for n = 17.

**Figure:**
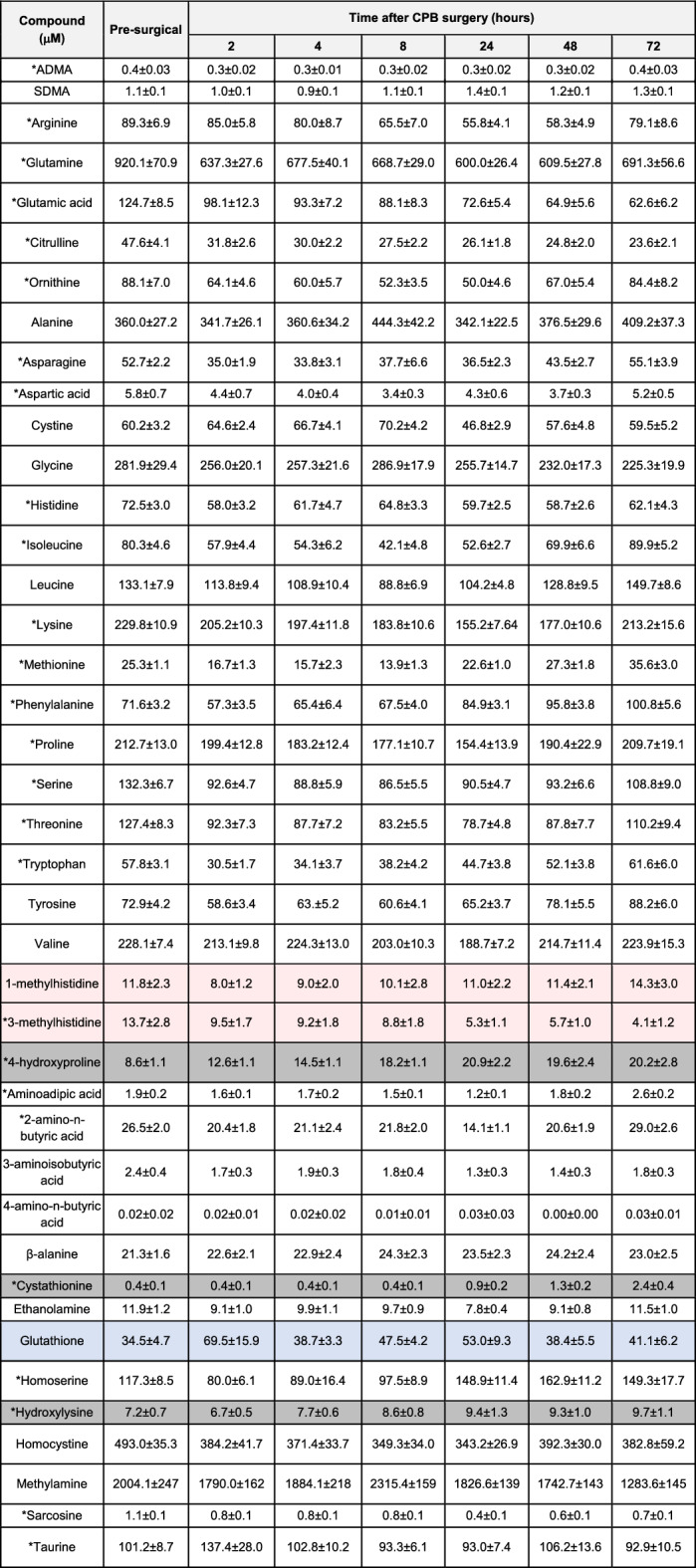

**Table S4:** List of 38 amines associated with regions A, B and C of ellipses, as according to PLS-DA scatter plot. Amines clustered around region A, region B and region C (see Supplementary Figure 3) of ellipses, generated from PLS-DA of 24-hour samples and pre-surgical sample group, are detailed in corresponding columns in table. Data are mean ± SEM for n = 17 patients who underwent CPB surgery and exhibited SIRS post-surgery.

Region A	Region B	Region C
4-hydroxyproline	Beta-alanine	ADMA
Cystathionine	Methylamine	Arginine
SDMA	Glycine	Glutamine
Hydroxylysine	Taurine	Glutamic acid
Phenylalanine	Alanine	Citrulline
Homoserine	Tyrosine	Ornithine
		Asparagine
		Aspartic acid
		Cystine
		Histidine
		Isoleucine
		Leucine
		Lysine
		Methionine
		Proline
		Serine
		Threonine
		Tryptophan
		Valine
		Aminoadipic acid
		2-amino-n-butyric acid
		3-amino-iso-butyric acid
		4-amino-n-butyric acid
		Ethanolamine
		Homocystine
		Sarcosine

