# Supplementary material for: Development of a novel UHPLC-MS/MS-based platform to quantify amines, amino acids and methylarginines for applications in human disease phenotyping
Source: Sci Rep. 2018 Sep 18;8:13987. doi: 10.1038/s41598-018-31055-8 (PMC6143519; doi:10.1038/s41598-018-31055-8)
Supplement: Supplementary file 1 — Supplementary information [file 41598_2018_31055_MOESM1_ESM.pdf]

Development of a novel UHPLC-MS/MS-based platform to quantify amines, amino acids and methylarginines for applications in human disease phenotyping

Blerina Ahmetaj-Shala[1*], Michael Olanipekun[2*], Abel Tesfai[1*], Niall MacCallum[3], Nicholas S Kirkby[1], Gregory J Quinlan[1], Chih-Chin Shih[1], Ryota Kawai[1], Sharon Mumby[4], Mark Paul-Clark[1], **Elizabeth Want[2#] and Jane A Mitchell[1#]**

Supporting information

Supplementary Figure 1. PCA-X and PLS-DA score plots for all patients at every time point, including previously excluded amines 1-methylhistidine, 3-methylhistidine and glutathione. (A) The PCA-X scatter plot of all amine peak areas obtained from plasma samples collected before (0) and after (2-72 hours) surgery. Observations are shown as the patient identifiers at each time point and are scattered based on the data for 42 compounds detected using UHPLC-MS/MS. Data within the ellipse represents the tolerance of Hotelling's T^2, revealing outliers as observations present outside of this area. **(B)** The PLS-DA scatter plot shows the deviation of the 24-hour samples (blue) from the pre-surgical sample group (green) via their separate clustering. **(C)** The contributions of each amine to the deviation is represented by the contribution plot, describing the change in amine levels from the pre-surgical (green) to the 24-hour (blue) samples. These changes were assessed based on the weighted differences between the datasets (w*1w*2) in the PLS-DA model. Data shown is for n=17.

Supplementary Figure 2. Effects of ADMA and L-NMMA on iNOS activity in LPS activate J774 mouse macrophages. Cells were stimulated with LPS in the presence of drugs for 24 h. Data is mean ± SEM for n=4 separate experiments.

Supplementary Figure 3. A schematic of PLS-DA ellipse split into region associated with 24 h samples (region A), region not associated with either time points (region B) and region associated with 72 h (region C).

Supplementary Table 1. Chromatographic and mass spectrometric characteristics of the analytes measured using UHPLC-MS/MS. Individual parameters for the amines were determined using the UHPLC-MS/MS instrument. Each amine was allocated a unique ID.

Supplementary Table 2. CPB patient demographics. Demographics of the 18 patients undergoing CPB surgery, 17 of which met the SIRS criteria and had a complete set of samples for all investigated time points and 1 of which did not (highlighted in grey). (AVR= aortic valve replacement, CABG= coronary artery bypass graft, MVR= mitral valve replacement, M= male, F= female).

Supplementary Table 3. List of 42 amines measured in human plasma using UHPLC-MS/MS. Data are mean ± SEM for n=17 patients who underwent CPB surgery and exhibited SIRS post-surgery. Data were analysed by repeated measures one-way ANOVA with Dunnett's post-hoc test (*$p<0.05$ when compared to individual levels pre-surgery). Amines significantly increased are highlighted in grey. Amines highlighted in pink were processed without internal standards to achieve a passable standard curve, while glutathione, highlighted in blue, did not have an acceptable standard curve ($r^2<0.95$).

Supplementary Table 4. List of 39 amines associated with regions A, B and C of ellipses, as according to PLS-DA scatter plot. Amines clustered around region A, region B and region C (see Supplementary Figure 3) of ellipses, generated from PLS-DA of 24-hour samples and pre-surgical sample group, are detailed in corresponding columns in table. Data are mean ± SEM for n=17 patients who underwent CPB surgery and exhibited SIRS post-surgery.

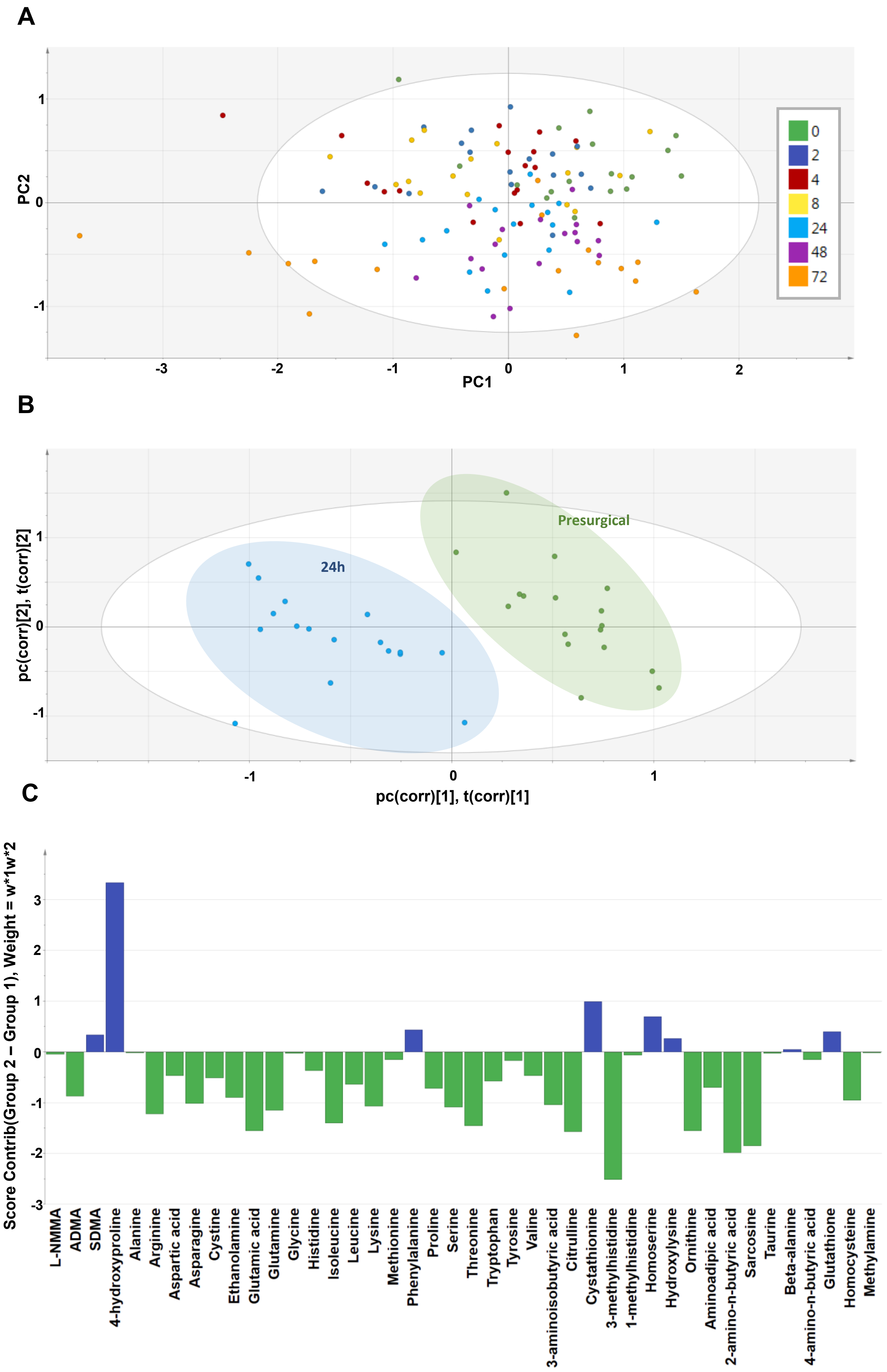

Supplementary Figure 1

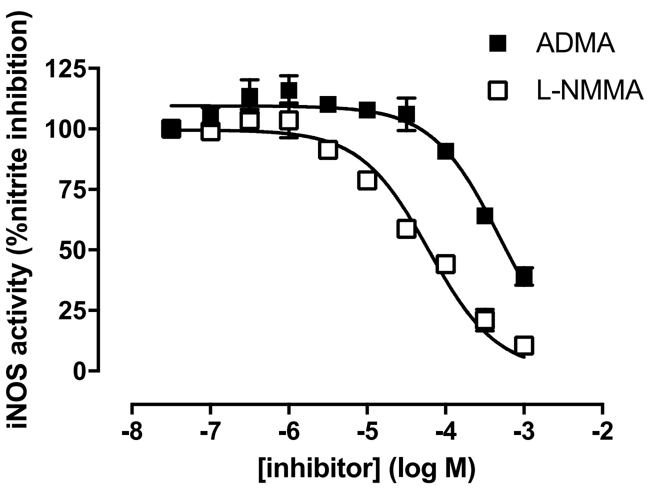

Supplementary Figure 2.

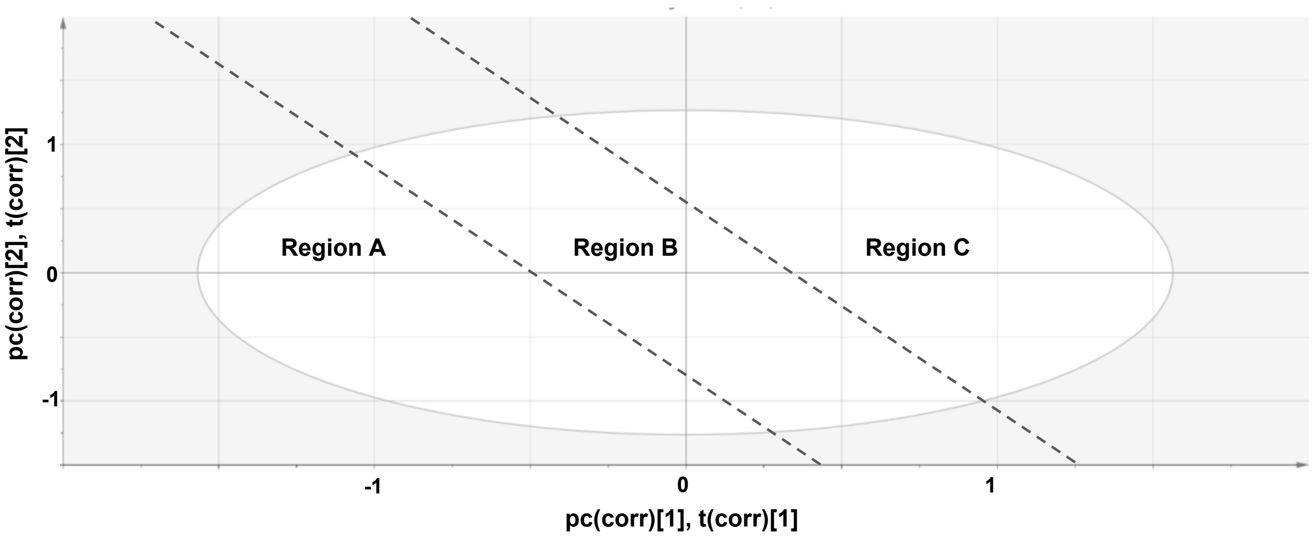

Supplementary Figure 3

Compound	Parent (m/z)	Fragment (m/z)	Window (min)	Dwell time (s)	Cone voltage (V)	Collision energy (eV)	RT (min)
ADMA	373.2	158.0	2.49-2.89	0.009	30	16	2.67
L-NMMA	359.0	188.9	2.42-2.82	0.009	30	26	2.62
SDMA	373.2	172.0	2.67-3.07	0.009	30	22	2.87
Arginine	345.1	70.1	1.80-2.20	0.009	30	36	2.09
Glutamine	317.1	171.1	2.00-2.50	0.009	30	24	2.26
Glutamic acid	318.1	171.1	2.50-3.20	0.011	30	22	2.94
Citrulline	346.2	171.1	2.50-2.90	0.011	30	26	2.75
Ornithine	303.1	171.1	3.60-4.20	0.009	60	22	3.86
Alanine	260.1	116.1	2.60-3.60	0.011	30	44	3.43
Asparagine	303.1	171.1	1.64-2.04	0.009	30	22	1.97
Aspartic acid	304.1	171.1	2.56-2.96	0.011	30	22	2.65
Cystine	291.1	171.1	3.92-4.32	0.009	10	12	4.08
Glycine	246.1	116.1	2.20-2.80	0.011	30	40	2.56
Histidine	326.1	156.1	0.00-2.00	0.011	20	10	1.55
Isoleucine	302.1	171.1	5.00-5.50	0.024	30	20	5.22
Leucine	302.1	171.1	5.00-5.50	0.024	30	20	5.26
Lysine	244.2	171.1	4.00-4.40	0.020	30	12	4.10
Methionine	320.1	171.1	4.30-4.80	0.017	30	22	4.59
Phenylalanine	336.1	171.1	5.20-5.70	0.024	30	22	5.46
Proline	286.1	116.1	3.50-4.00	0.009	30	50	3.79
Serine	276.1	171.1	2.00-2.50	0.009	30	20	2.28
Threonine	290.1	171.1	2.85-3.25	0.018	30	20	3.07
Tryptophan	375.1	171.1	5.20-5.80	0.024	30	26	5.51
Tyrosine	352.1	171.1	4.20-4.80	0.017	30	24	4.41
Valine	288.1	171.1	4.40-4.80	0.017	30	16	4.64
1-methylhistidine	340.1	124.2	0.00-2.20	0.009	30	28	1.84

3-methylhistidine	340.1	170.1	0.00-2.20	0.009	30	18	1.73
4-hydroxyproline	302.0	171.1	1.50-2.00	0.011	10	22	1.75
Aminoadipic acid	332.1	171.1	3.20-3.80	0.011	30	18	3.59
2-amino-n-butyric acid	274.1	171.1	3.20-4.20	0.009	10	20	4.07
3-aminoisobutyric acid	274.1	171.1	3.20-4.20	0.009	10	20	3.77
4-amino-n-butyric acid	274.1	171.1	3.20-4.20	0.009	10	20	3.50
β-alanine	260.1	171.1	2.60-3.60	0.011	30	20	3.04
Cystathionine	282.2	171.0	3.80-4.20	0.009	30	14	3.85
Ethanolamine	232.1	171.1	2.20-2.70	0.011	10	20	2.43
Glutathione	478.0	171.1	3.57-3.97	0.009	30	38	3.76
Homoserine	290.1	171.1	2.20-2.60	0.013	10	18	2.42
Hydroxylysine	252.1	171.0	3.45-3.85	0.011	30	28	3.59
Homocystine	439.1	171.1	3.40-5.10	0.009	10	24	4.87
Methylamine	202.1	171.1	2.56-2.96	0.010	30	16	1.63
Sarcosine	260.1	171.1	2.60-3.60	0.011	30	20	2.82
Taurine	296.1	116.3	2.00-2.50	0.009	30	60	2.18

Supplementary Table 1.

Patient (n)	Gender (M/F)	Age (years)	CPB Surgery type	SIRS ≥2 con 24	SIRS status (Y/N)
1	M	63	AVR	3	Y
2	M	44	AVR	5	Y
3	M	80	CABG	6	Y
4	F	76	AVR	1	Y
5	M	82	AVR	6	Y
6	M	65	CABG	7	Y
7	F	84	CABG	4	Y
8	M	73	CABG	5	Y
9	M	61	MVR	14	Y
10	F	70	AVR CABG	3	Y
11	M	49	CABG	4	Y
12	M	49	CABG	17	Y
13	M	69	CABG	1	Y
14	M	61	CABG	7	Y
15	M	55	CABG	1	Y
16	M	64	CABG	3	Y
17	F	74	AVR	3	Y
18	M	69	CABG	3	Y

Supplementary Table 2.

Compound (μM)	Pre-surgical	Time after CPB surgery (hours)					
		2	4	8	24	48	72
*ADMA	0.4±0.03	0.3±0.02	0.3±0.01	0.3±0.02	0.3±0.02	0.3±0.02	0.4±0.03
*L-NMMA	2.1±0.2	1.8±0.2	1.2±0.1	2.0±0.2	2.0±0.2	1.5±0.2	1.3±0.2
SDMA	1.1±0.1	1.0±0.1	0.9±0.1	1.1±0.1	1.4±0.1	1.2±0.1	1.3±0.1
*Arginine	89.3±6.9	85.0±5.8	80.0±8.7	65.5±7.0	55.8±4.1	58.3±4.9	79.1±8.6
*Glutamine	920.1±70.9	637.3±27.6	677.5±40.1	668.7±29.0	600.0±26.4	609.5±27.8	691.3±56.6
*Glutamic acid	124.7±8.5	98.1±12.3	93.3±7.2	88.1±8.3	72.6±5.4	64.9±5.6	62.6±6.2
*Citrulline	47.6±4.1	31.8±2.6	30.0±2.2	27.5±2.2	26.1±1.8	24.8±2.0	23.6±2.1
*Ornithine	88.1±7.0	64.1±4.6	60.0±5.7	52.3±3.5	50.0±4.6	67.0±5.4	84.4±8.2
Alanine	360.0±27.2	341.7±26.1	360.6±34.2	444.3±42.2	342.1±22.5	376.5±29.6	409.2±37.3
*Asparagine	52.7±2.2	35.0±1.9	33.8±3.1	37.7±6.6	36.5±2.3	43.5±2.7	55.1±3.9
*Aspartic acid	5.8±0.7	4.4±0.7	4.0±0.4	3.4±0.3	4.3±0.6	3.7±0.3	5.2±0.5
Cystine	60.2±3.2	64.6±2.4	66.7±4.1	70.2±4.2	46.8±2.9	57.6±4.8	59.5±5.2
Glycine	281.9±29.4	256.0±20.1	257.3±21.6	286.9±17.9	255.7±14.7	232.0±17.3	225.3±19.9
*Histidine	72.5±3.0	58.0±3.2	61.7±4.7	64.8±3.3	59.7±2.5	58.7±2.6	62.1±4.3
*Isoleucine	80.3±4.6	57.9±4.4	54.3±6.2	42.1±4.8	52.6±2.7	69.9±6.6	89.9±5.2
Leucine	133.1±7.9	113.8±9.4	108.9±10.4	88.8±6.9	104.2±4.8	128.8±9.5	149.7±8.6
*Lysine	229.8±10.9	205.2±10.3	197.4±11.8	183.8±10.6	155.2±7.64	177.0±10.6	213.2±15.6
*Methionine	25.3±1.1	16.7±1.3	15.7±2.3	13.9±1.3	22.6±1.0	27.3±1.8	35.6±3.0
*Phenylalanine	71.6±3.2	57.3±3.5	65.4±6.4	67.5±4.0	84.9±3.1	95.8±3.8	100.8±5.6
*Proline	212.7±13.0	199.4±12.8	183.2±12.4	177.1±10.7	154.4±13.9	190.4±22.9	209.7±19.1
*Serine	132.3±6.7	92.6±4.7	88.8±5.9	86.5±5.5	90.5±4.7	93.2±6.6	108.8±9.0
*Threonine	127.4±8.3	92.3±7.3	87.7±7.2	83.2±5.5	78.7±4.8	87.8±7.7	110.2±9.4
*Tryptophan	57.8±3.1	30.5±1.7	34.1±3.7	38.2±4.2	44.7±3.8	52.1±3.8	61.6±6.0
Tyrosine	72.9±4.2	58.6±3.4	63.±5.2	60.6±4.1	65.2±3.7	78.1±5.5	88.2±6.0
Valine	228.1±7.4	213.1±9.8	224.3±13.0	203.0±10.3	188.7±7.2	214.7±11.4	223.9±15.3
1-methylhistidine	11.8±2.3	8.0±1.2	9.0±2.0	10.1±2.8	11.0±2.2	11.4±2.1	14.3±3.0

*3-methylhistidine	13.7±2.8	9.5±1.7	9.2±1.8	8.8±1.8	5.3±1.1	5.7±1.0	4.1±1.2
*4-hydroxyproline	8.6±1.1	12.6±1.1	14.5±1.1	18.2±1.1	20.9±2.2	19.6±2.4	20.2±2.8
*Aminoadipic acid	1.9±0.2	1.6±0.1	1.7±0.2	1.5±0.1	1.2±0.1	1.8±0.2	2.6±0.2
*2-amino-n-butyric acid	26.5±2.0	20.4±1.8	21.1±2.4	21.8±2.0	14.1±1.1	20.6±1.9	29.0±2.6
3-aminoisobutyric acid	2.4±0.4	1.7±0.3	1.9±0.3	1.8±0.4	1.3±0.3	1.4±0.3	1.8±0.3
4-amino-n-butyric acid	0.02±0.02	0.02±0.01	0.02±0.02	0.01±0.01	0.03±0.03	0.00±0.00	0.03±0.01
β-alanine	21.3±1.6	22.6±2.1	22.9±2.4	24.3±2.3	23.5±2.3	24.2±2.4	23.0±2.5
*Cystathionine	0.4±0.1	0.4±0.1	0.4±0.1	0.4±0.1	0.9±0.2	1.3±0.2	2.4±0.4
Ethanolamine	11.9±1.2	9.1±1.0	9.9±1.1	9.7±0.9	7.8±0.4	9.1±0.8	11.5±1.0
Glutathione	34.5±4.7	69.5±15.9	38.7±3.3	47.5±4.2	53.0±9.3	38.4±5.5	41.1±6.2
*Homoserine	117.3±8.5	80.0±6.1	89.0±16.4	97.5±8.9	148.9±11.4	162.9±11.2	149.3±17.7
*Hydroxylysine	7.2±0.7	6.7±0.5	7.7±0.6	8.6±0.8	9.4±1.3	9.3±1.0	9.7±1.1
Homocystine	493.0±35.3	384.2±41.7	371.4±33.7	349.3±34.0	343.2±26.9	392.3±30.0	382.8±59.2
Methylamine	2004.1±247	1790.0±162	1884.1±218	2315.4±159	1826.6±139	1742.7±143	1283.6±145
*Sarcosine	1.1±0.1	0.8±0.1	0.8±0.1	0.8±0.1	0.4±0.1	0.6±0.1	0.7±0.1
*Taurine	101.2±8.7	137.4±28.0	102.8±10.2	93.3±6.1	93.0±7.4	106.2±13.6	92.9±10.5

Supplementary Table 3.

Region A	Region B	Region C
4-hydroxyproline	Beta-alanine	ADMA
Cystathionine	Methylamine	Arginine
SDMA	Glycine	Glutamine
Hydroxylysine	Taurine	Glutamic acid
Phenylalanine	Alanine	Citrulline
Homoserine	L-NMMA	Ornithine
	Tyrosine	Asparagine
		Aspartic acid
		Cystine
		Histidine
		Isoleucine
		Leucine
		Lysine
		Methionine
		Proline
		Serine
		Threonine
		Tryptophan
		Valine
		Aminoadipic acid
		2-amino-n-butyric acid
		3-amino-iso-butyric acid
		4-amino-n-butyric acid
		Ethanolamine
		Homocystine
		Sarcosine

Supplementary Table 4.
